# Stable Odor Recognition by a neuro-adaptive Electronic Nose

**DOI:** 10.1038/srep10960

**Published:** 2015-06-04

**Authors:** Eugenio Martinelli, Gabriele Magna, Davide Polese, Alexander Vergara, Detlev Schild, Corrado Di Natale

**Affiliations:** 1 Department of Electronic Engineering, University of Rome Tor Vergata, Via del Politecnico 1, Rome 00133, Italy; 2 BioCircuits Institute, University of California San Diego, 9500 Gilman Dr., La Jolla, CA 92093-0402, USA; 3 Inst. of Neurophysiology and Cellular Biophysics, University of Göttingen, Humboldtallee 23, 37077 Göttingen, Germany; 4 DFG Excellence Cluster 171 and Bernstein Forum of Neurotechnology, Univ. Göttingen.

## Abstract

Sensitivity, selectivity and stability are decisive properties of sensors. In chemical gas sensors odor recognition can be severely compromised by poor signal stability, particularly in real life applications where the sensors are exposed to unpredictable sequences of odors under changing external conditions. Although olfactory receptor neurons in the nose face similar stimulus sequences under likewise changing conditions, odor recognition is very stable and odorants can be reliably identified independently from past odor perception. We postulate that appropriate pre-processing of the output signals of chemical sensors substantially contributes to the stability of odor recognition, in spite of marked sensor instabilities. To investigate this hypothesis, we use an adaptive, unsupervised neural network inspired by the glomerular input circuitry of the olfactory bulb. Essentially the model reduces the effect of the sensors’ instabilities by utilizing them via an adaptive multicompartment feed-forward inhibition. We collected and analyzed responses of a 4 × 4 gas sensor array to a number of volatile compounds applied over a period of 18 months, whereby every sensor was sampled episodically. The network conferred excellent stability to the compounds’ identification and was clearly superior over standard classifiers, even when one of the sensors exhibited random fluctuations or stopped working at all.

Over the last two decades the development and performance of electronic noses has seen enormous progress in many respects^1^,^2^. However, the chemical sensors have changed surprisingly little. In most cases their output signals are still notoriously unstable, particularly when stimuli are presented rarely, intermittently with others, and over a long period of time^3^. The sensors are currently operated as open-loop systems in which the interaction occurring at their surface is converted into a read-out signal. From an electronics point of view gas sensitive resistors such as metal-oxide semiconductors are simple devices but they can show a rather complex behavior. For most stimuli the individual sensors of a sensor array show overlapping odor selectivity spectra, and, in addition, their outputs heavily depend on a number of ancillary parameters like the partial oxygen pressure at the surface, the local surface temperature, and the interference of concurrent molecules such as water and/or oxidizing or reducing compounds^4^. Some of these parameters are difficult to control even in the laboratory, and most of them are beyond control in real life applications. Even in cases where the causes of such error sources were known, it would be difficult if not impossible to predict their occurances in time and their intensities.

Similar issues are known in olfactory receptor neurons (ORNs)^5^,^6^, and it was this parallel that suggested a strategy how to deal with instability and non-reproducibility of sensor signals^7^,^8^. Besides of trying to improve sensors or their usage, we sought to compensate sensor fluctuations by using an appropriate adaptive neural network.

There are a number of noteworthy parallels between chemical sensors and ORNs^9^,^10^. In contrast to all other neurons ORNs are exposed directly to the body environment as their cilia protrude into the mucus of the olfactory epithelium^5^. This may be one reason why they are continually replaced. ORNs are also known to show fluctuations in their stimulus responses when repeatedly presented with the same stimulus^11^. Responses to stimuli that are intermittently presented over many hours have not been measured so far, and recordings over much longer periods are impossible, since the cells die after a few weeks, are replaced, and the newborn ORNs are then rewired to the subsequent stage of signal processing, i.e., the glomeruli of the olfactory bulb^12^. Specific connectivities must therefore be re-established, which is supposed to be another source of fluctuation.

Despite the complex dynamics at the stage of individual receptors, the *ensemble* of ORNs although undergoing continuous regeneration and rewiring to the olfactory bulb appears to provide a reliable olfactory perception. Learnt odors can be recognized after very long periods of time with high fidelity and animals with severe olfactory lesions (e.g., after removal of 20% of the one type of genetically defined olfactory receptor neurons) retain the remarkable capability to accurately recognize and distinguish odorant molecules^13^,^14^.

This study includes a number of features of the olfactory system. First, we use a 4 × 4 - sensor array comprising four sensor classes and four sensors of each class, corresponding to four ORN classes and four cells per class. Second, we mimic the connectivity of ORNs to the olfactory bulb by using an adaptive multi-compartment glomerular model that takes into account nonlinear inhibition between periglomerular cells and mitral cells^15^. Third, we tried to come close to natural odor application schemes. After the glomerular network was trained to the gases used in this study, these were presented over a long time (18 months) in a random sequence and in bursts of a few applications with long periods in between during which either other gases or no stimuli at all were measured. Finally, we investigated how the network reacted to sensor failures, in particular to a random input or the total drop-out of a sensor.

To assess the odor discrimination performance of the adaptive network, we classified the network’s output using Partial Least Squares Discriminant Analysis (PLS-DA)^16^, and compared the outcome to the direct PLS-DA evaluation of the sensor array output.

Our results demonstrate that an adaptive multi-compartment network mimicking features of periglomerular cells can largely compensate sensor signal fluctuations and thereby markedly improve stimulus recognition in an electronic nose.

## Results

**Figure 1:**
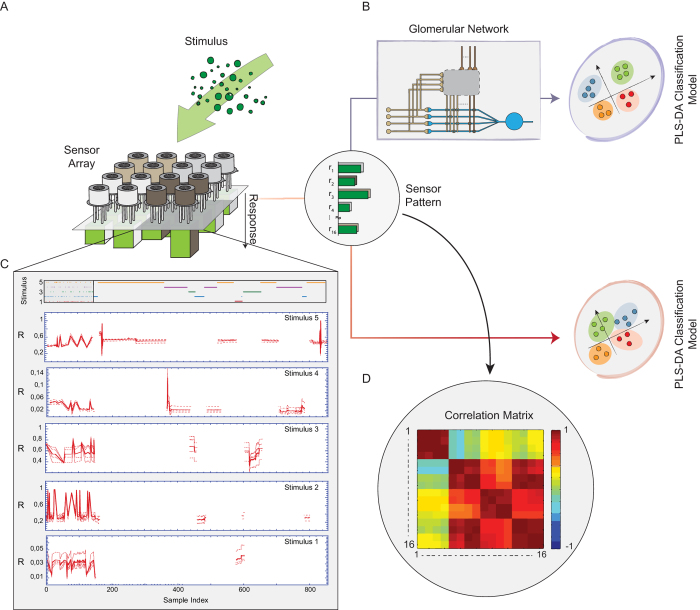
Gas detection using a chemical sensor array and two evaluation methods. **A**) 4 × 4 array of chemical sensors. Four sensor classes and 4 sensors per sensor class. **B**) The pattern vector of the 16 sensor outputs, r_1_ through r_16_, are fed in a neural network (upper branch) and then analyzed by the PLS-DA algorithm. For comparison the same data are analyzed without the network (lower branch). **C**) Example of responses of one sensor class to the five odorants. Upper panel, sequence of stimuli applied. Subsequent panels, overview over the responses of the four sensors of one class to the five stimuli, the average being drawn in bold. Note the prominent jumps upon some stimulus changes. **D**) Correlation matrix of sensor array response vector in the training phase.

A 4 × 4 array of chemical sensors was used for the detection of five volatile organic compounds (VOCs, Fig. 1A, Methods). The sensor signals were fed into a bio-inspired network, the output of which was then analyzed using a standard classification algorithm, PLS-DA (Fig. 1B, upper). The overall performance of the network/PLS-DA was compared to an analysis of the same signals by the PLS-DA algorithm alone (Fig. 1B, lower). The lengths of the stimulus application episodes differed from stimulus to stimulus (Suppl. Fig. 1).

### Chemical sensor array output

The responses of one of the sensor classes to the five stimuli used are shown Fig. 1C, where the four sensor signals (dotted) were plotted together with their average (solid) for each stimulus. For instance, acetone (stimulus #3) and ethanol (stimulus #5) were applied for two and four episodes, respectively. In the inter-episode gaps other stimuli were applied.

The time courses of the individual sensor signals are characterized by large fluctuations, mostly drift and jumps, both within and in between episodes, whereby the individual sensors behaved in a similar way. The corresponding data for all sensor classes are given in Suppl. Fig. 2.

In accord with the correlated fluctuations, the correlation matrix of the 16 sensor output channels shows, in addition to the expected high values for the sensors of the same class, also marked correlations among sensors of different classes. In particular class 2, class 4, and, albeit to a lesser extent, class 3 are highly correlated (Fig. 1D). The sensor array used in this study thus appears to be far from ideal for distinguishing the five compounds.

**Figure 2:**
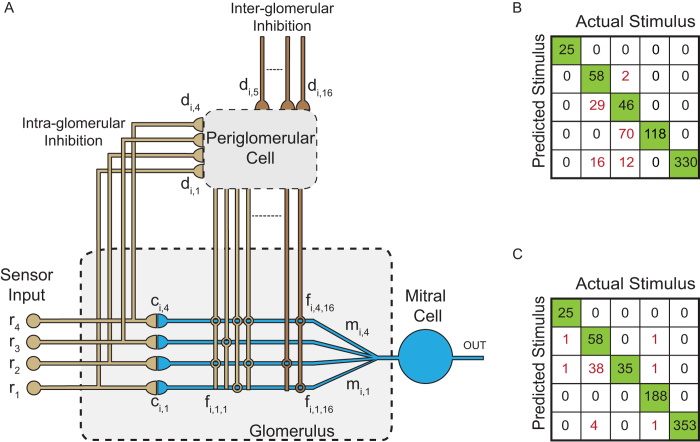
Adaptive neural network and its effect on VOCs classification. **A**) Shown is the first mitral cell, which is connected to the sensor output signals of the first sensor class, i.e., r_1_, r_2_, r_3_, r_4_, via weights c_1j_. The same outputs excite the first periglomerular (PG) cell via weights d_1ki_. The PG cell received additional inputs from all other sensor classes. Each input of the PG cell can inhibit the mitral cell input branches. **B**) Confusion matrix as resulting from the PLS-DA alone (testing phase). **C**) Confusion matrix as resulting from the adaptive network with subsequent PLS-DA after one hundred simulations (testing phase).

In fact, applying PCA to the sensor outputs (Suppl. Fig. 3) revealed only ammonia as clearly distinct from the other compounds, whereas the first two principal components of the other stimuli clearly overlap. It was thus not unexpected that the PLS-DA of the sensor signals led to a rather inhomogeneous confusion matrix (Fig. 2B) with large errors occurring for compound #3 (38.7%) and #4 (37%). Thus, the rate of correct classifications of 82% was unsatisfactory not only because of the number itself but also because it was brought about by largely differing classification rates.

We considered that averaging the sensor signals within each class prior to PLS-DA might improve the analysis, but the contrary turned out to be the case. When the averages of the four sensor signals of every sensor class were fed into the PLS-DA, the outcome was either perfect recognition (compound #1,#2, and #4), complete failure (compound #3), or close to chance (compound #5, Fig. 2B). Averaging sensor signals prior to PLS-DA was thus not advantageous, mostly because averaging does not smooth correlated fluctuations.

To improve the classification performance it therefore seemed to be more promising to counteract the effects of jumps and drift by using the correlated signals themselves. Preliminary tests suggested to adopt a neuronal network which essentially modulates the effect of individual inputs as a function of all inputs^15^.

### Adaptive neuronal network

We fed the sensor outputs into a neuronal network with self-adapting weights (Fig. 1B, Fig. 2A, Methods) before applying the PLS-DA classifier. Specifically, we connected the four outputs of the i-th sensor class to the i-th MC ,while the associated i-th PG cell was allowed to receive input from all sensors (Fig. 2A). The initial connectivity was random and the learning parameters were taken from the original paper^15^. Under these conditions, the average rate of correct classifications calculated from one hundred trials using different initial conditions was approximately 90%, whereby, importantly, the precision was excellent, the classification error per stimulus mostly being in the range of or lower than 1% as shown in the corresponding confusion matrix (Fig. 2C). Hence, not only did the pre-processing by the adaptive network improve the correct classification rates, but each stimulus was also reliably classified with high precision (Fig. 2B,C).

### Compensation of sensor faults

External circumstances and limited lifetime compromise chemical sensors as well as olfactory receptor neurons. It was hence intriguing to see how the network responded to the fault of individual sensors. We simulated the failure of a sensor and its later re-appearance, corresponding to death and regeneration of an olfactory receptor neuron. Two cases were considered: complete loss of function (sensor output r = 0) or random fluctuations (random r with mean 0.5).

**Figure 3:**
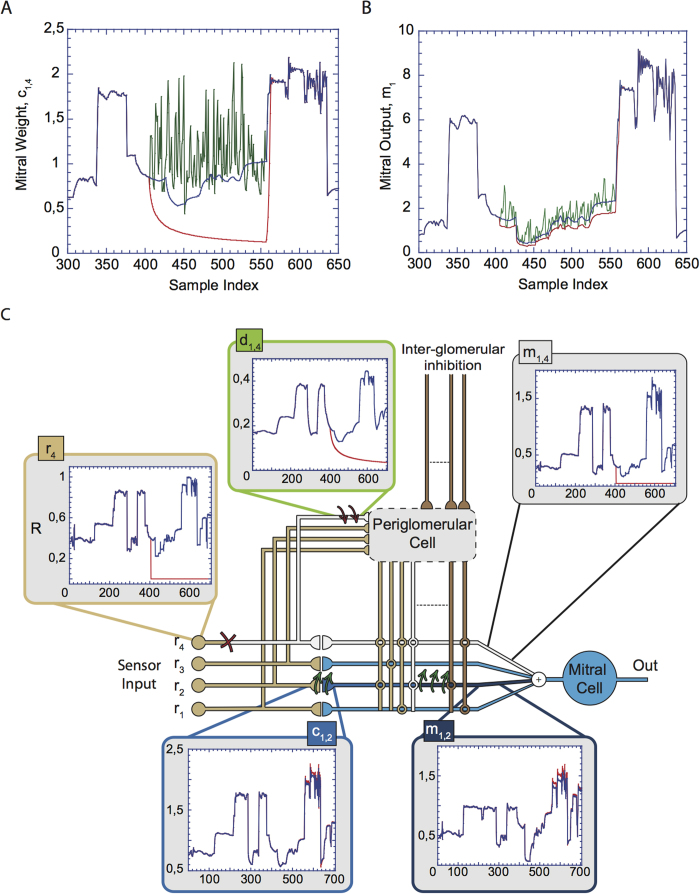
Compensation of sensor fault. The 4^th^ sensor of the first sensor class, i.e. r_4_, is assumed to go into fault between sample index 400 and sample index 520. The red and green traces refer to a total drop-out (red) and to a randomly fluctuating sensor output r_4_, respectively, while the blue trace shows the behavior without fault. **A**) Decay of the synaptic weight c_14_ between 4^th^ sensor of class 1 and first mitral cell (red) or random fluctuations of c_14_ (green). **B**) Corresponding output of MC #1. **C**) System variables of the first glomerulus for a longer fault period. Upon fault of r_4_, d_14_ behaves similarly to c_14_ (A). The affected MC branch, having no input, follows the sensor input immediately. Other MC branches such as m_21_, being normally inhibited by the PG cell via r_4_, are now disinhibited and strengthen the input connection c_12_.

The trial shown in Fig. 3A exemplifies the effects of a sensor fault upon the coupling (synaptic strength c_ij_) to the mitral cell affected. It either drops, first rapidly and then very slowly, to zero, or it shows large random fluctuations during the fault. Despite these dramatic changes of one network input, the output of the affected glomerular unit closely followed the control trace (Fig. 3B), albeit with smaller amplitudes (red) or random noise (green). After re-attaching the proper sensor output to the preprocessor, the MCs output rapidly converged to the time-course it took without fault (Fig. 3B).

The effects of a total drop-out of one sensor are summarized in Fig. 3C. The left panel in the middle shows the responses of the 4^th^ sensor of sensor class 1, r_4_, along with the stimulus input pattern. Expectedly, r_4_ responds differentially to the subsequent stimuli. At a certain point (sample index: 400) the input is set to zero (red). The immediate consequences are (i) that the potential on the corresponding postsynaptic MC branch (m_14_) vanishes (upper right panel), and that (ii) c_14_ (Fig. 3A) and d_14_ (upper center panel) tend to zero in a characteristic way, first rapidly and then much more slowly due to the non-linear learning rule (eq. 3). The resulting effect is a dis-inhibition of the affected remaining MC inputs. E.g., the inhibition which r_4_ normally exerts upon m_12_ is lacking under the fault leading to an increase of m_12_ (lower right) and a subsequent strengthening of the c_12_ connection (lower left). In addition, the drop-out of r_4_ can disinhibit other MCs if connected to their PG cells.

**Figure 4:**
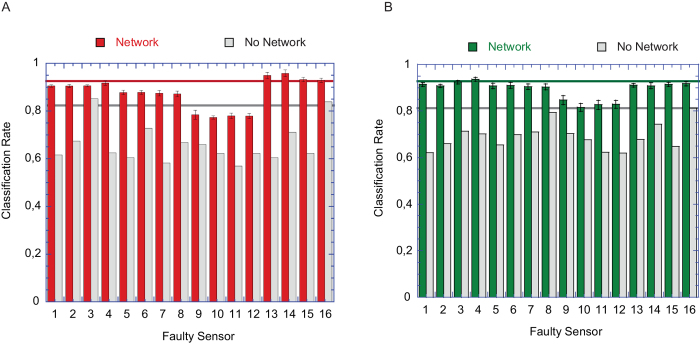
Rates of correct classification for two fault conditions. **A**) Correct classifications with adaptive network (red) or without adaptive network (gray) depending on which sensor exactly was in fault. Red and gray horizontal lines are the control cases (i.e., without fault) and show the average correct classification rates with adaptive network (red) or without adaptive network (gray). **B**) Same as in A but for one randomly fluctuating sensor output. Note that in cases where the faulty sensor contributed more noise than the other sensors, the classification rate of the network-PLS-DA can exceed the maximum.

The effect of a sensor fault upon the network’s output also depended on which sensor exactly was faulty and how it was connected to the network. Fig. 4 shows the correct classification rates of both the direct PLS-DA (gray) and the adaptive network plus PLS-DA (color), first, for the case of complete loss of sensor function (Fig. 4A) and second, for the case of one random sensor signal (Fig. 4B). Though generally the correct classification rates decreased during a fault, the adaptive network always performed better than the PLS-DA alone. In many cases its performance was close to or indistinguishable from the control classification performance where all sensors were intact (Fig. 4, colored or gray lines, respectively).

## Discussion

Our understanding of odor discrimination mechanisms in biology has stimulated the development of a variety of models and algorithms that have been applied in a number of application areas^17^,^18^,^19^. The experiments reported in this paper differ from these studies in that we focus on the compensation of input instabilities taking into account a number of features of the olfactory system. First, olfactory receptor neurons fall into classes each of which expresses the same olfactory receptor^12^. So we choose a 4 × 4 - sensor array with four sensor classes and four sensors per class. Second, olfactory receptor neurons of the same class converge to the same glomerulus^12^. Thus, we stipulated the connectivities c_ij_ to be zero for all other ORN - MC projections (see explanation of eq. 2 in Methods). Third, ORNs in the olfactory epithelium undergo a continuous regeneration and turn-over. In analogy, we allowed dynamic adaptation of all synaptic weights, not only in an initial phase of the experiments. Finally, from the few studies on PG cells we extracted two common denominators, i.e., PG cells receive input from ORNs and can inhibit the ORN input to MCs^20^, whereby anaxonic PG cells are presumably responsible for intraglomerular inhibition^21^. Although the inhibition of MCs by sensor/receptor neurons might at first glance be reminiscent of rotating the connectivity matrix between ORNs and MCs, the PG cell - mediated effects of our network are different. First, they are exclusively inhibitory and thus not corresponding to a linear matrix rotation. Second, the synaptic weights decay in a nonlinear and self-organized way, which may decorrelate inputs more effectively than a linear operation would^22^,^23^,^24^. Finally, the nonlinearity may be important for small synaptic weights, which decay more slowly than in the linear case and can therefore more easily recover in case of a changing environment^15^.

In comparison to other models, the large number of parameters of our adaptive model might be seen as a disadvantage, but it appears that the ORN-specific inhibition of MC input branches by PG cells is particularly important and must not be reduced or pooled, if input fluctuations are to be dampened. In the same context, it had to be expected that averaging sensor signals prior to classifying stimuli was not advantageous. Averaging is known to smooth only in case of uncorrelated fluctuations, whereas the most pronounced fluctuations in the chemical sensor outputs were correlated.

In this study we tried to keep the complexity of our system and thus the number of its parameters as low as possible. As a consequence we chose to test all odorants at one concentration. Under more natural conditions the odorants’ concentrations could vary over a wide range, which might in some cases require a larger sensor array. In the context of concentration coding it would also be intriguing to analyze time-dependent response spectra with component-dependent delays brought about at low concentrations^25^,^26^. This analysis was however beyond the scope of the present study. Taken together, the redundant convergence from sensors to MCs together with the nonlinear adaptive network shaping the ORN-to-MC connectivity appears to confer stability to the network’s output, in spite of drifting and fluctuating inputs. Both features should thus be considered in the design of electronic noses. In addition, the network’s behavior in case of a faulty sensor would allow replacing faulty sensors on the fly without stopping ongoing measurements.

## Methods

### Stimuli and stimulus application

Five volatile compounds: ammonia, acetaldehyde, acetone, ethylene, and ethanol were used as stimuli. They were individually mixed with a flow of synthetic air (10% relative humidity) and administered for 90 s at a concentration of 225 ppm. After every measurement the system was cleaned with synthetic air for 200 s.

Prior to the measurements the network was trained by applying five stimuli, ammonia, acetaldehyde, acetone, ethylene and ethanol, individually and in rapid, irregular sequence, recording the responses and feeding them into the network. During this training period the five stimuli were applied, in total, 40, 50, 20, 20, and 20 times, respectively (Suppl. Fig. 1, left). After the training phase the sensor array was probed in more extended episodes of repeated applications of the same stimulus, whereby the number of episodes, the number of stimulus applications per episode and the type of stimulus varied irregularly (Suppl. Fig. 1). Specifically, the time intervals between episodes ranged from two weeks to two months and measurements of episodes alternated with other experiments. The number of repetitions of the same stimulus varied between 1 and 201, and the individual VOCs as listed above were applied 25, 60, 75, 188, and 358 times.

### Sensors and sensor signals

In analogy to the redundancy of olfactory receptor neurons in the nose we used a 4 × 4 sensor matrix consisting of four types or classes of commercial tin-oxide gas sensors (Figaro Inc.^27^) and four replicas of each sensor class (the serial numbers of the four classes were TGS2600, TGS2602, TGS2610, TGS2620). Details regarding the experimental setup have been previously described^28^. Briefly, the sensors were placed in a 60 mL Teflon/stainless steel cell connected in series to a vapor delivery system, which was controlled by a set of mass-flow controllers. The sensors were exposed to a flow of 200 mL/min over the whole length of the experiment and kept at 400 °C, which is the mid-point of the operational temperature range suggested by the manufacturer. The difference in resistance, ∆R, before and at the end of every stimulus exposure was taken as output signal.

The sixteen sensor signals were either analyzed directly using a PLS-DA algorithm^16^ or fed into an adaptive multi-compartment network^15^ (see below, Fig. 1B). Both were implemented in MATLAB (Mathworks).

### Bio-inspired pre-processing unit

The neural network adopted here was originally designed to explain the high information throughput through the glomerular layer of the olfactory bulb^15^. It incorporates a number of putative processing features in glomeruli and comprises two kinds of units corresponding to periglomerular (PG) and mitral cells (MC) (Fig. 2A). Here we assume an equal number (L) of MCs and PG cells and define a *glomerular unit to* consist of (i) a MC, (ii) a PG cell, (iii) the input synapses connecting to them, and (iv) the synapses among MCs- and PG cells. As we use a 4 × 4 sensor array, we consider the special cases of L = 4. We further follow the assumption^15^ that the sensor signals couple to dendritic subcompartments of both MC and PG cells through coupling coefficients (i.e., synaptic strengths) c_ij_ and d_ik_, respectively. Specifically, the output of the j-th sensor, r_j_, generates in the first place, a potential m_ij_ on a subcompartment of the i-th MC. Likewise, the output of the k-th sensor, r_k_, generates a potential p_ik_ in the k-th subcompartment of the i-th PG cell. In addition, we take into account that the local PG potentials p_ik_ can inhibit the MC compartments through the coefficients f_ijk_ (inhibitory synaptic strengths), resulting in the following equations for the dendritic subcompartments of MC and PG cells:


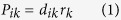



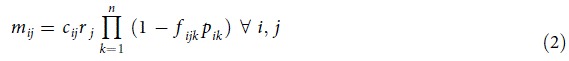


Hence, the output signal of the i-th MC is


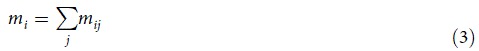


Sensor signals are indexed by two indices, j and k, in order to permit that the k-th sensor signal projecting to a PG cell may inhibit the effect of the j-th sensor cell. The total inhibition in each dendritic MC - branch is modeled as the product of several inhibition terms. Accordingly the network can provide strong inhibition through moderate values of f_ijk_ and p_ik_. It is interesting to note here that the inhibition on a MC - cell branch does not depend on whether the inhibition, i.e., the factor of the bracket in eq. 2, acts pre-synaptically (on the r’s) or post-synaptically (on the c’s).

It is generally accepted for an adult olfactory system that receptor neurons of the same class project into the same glomerulus. We take this convergence pattern into account by coupling the four replica signals of the first sensor class to the first glomerular unit, the second four signals to the second glomerular unit, etc., and set all other cij to zero, i.e. cij = 0 for j > 4, Fig. 2A). Taken together, the index i indicates both the i-th sensor class as well as the i-th glomerular unit and the i-th MC/PG cell complex. The index j stands for the j-th replica signal of a sensor class and the j-th input branch of the MC it projects to. The index k runs from 1 to 16 and indicates the sensor signal that projects to a PG cell.

The adaptation, which the network undergoes over the whole duration of the experiment, is controlled by the following rules for the synaptic weights c_ij_ and d_ik_


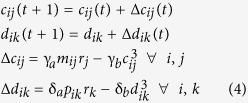


The coefficients γ_a_, γ_b_, δ_a_, δ_b_ define the synaptic learning speeds. In all simulations we have adopted the following values: γ_a_ = 5*10^−0.7^, γ_b_ = 10^−0.7^, δ_a_ = 10^−0.6^, δ_b_ = 5*10^−0.6^. The model implements a ‘local’ and nonlinear version of the Hebbian rule in that the dynamics of the synaptic weights c_ij_ and d_ij_ depend on the local dendritic potentials of MC- and PG cell micro-branches rather than on the cell outputs m_i_ or p_i_. This way the rule does generally not imply the correlation of one input with the others.

At the beginning of the experiment the values of the synaptic weights between sensors and MCs (c_ij_) and sensors and PG cells (d_ik_) were initialized randomly in (0,1), and each MC unit received input from one class of sensor, only. By contrast, PG cells could receive input from any type of input. The initial inhibitory connections between periglomerular and mitral cells (f_ijk_) were randomly chosen from (0, 0.1) and then randomly weighted with 0 or 1, i.e., whether or not an inhibitory connection between a specific PG and a specific mitral cell existed was assumed to be random.

## Additional Information

**How to cite this article**: Martinelli, E. *et al*. Stable Odor Recognition by a neuro-adaptive Electronic Nose. *Sci. Rep*. **5**, 10960; doi: 10.1038/srep10960 (2015).

## Supplementary Material

Supplementary Information
