# Supplementary material for: Stable Odor Recognition by a neuro-adaptive Electronic Nose
Source: Sci Rep. 2015 Jun 4;5:10960. doi: 10.1038/srep10960 (PMC4455291; doi:10.1038/srep10960)
Supplement: Supplementary Information [file srep10960-s1.doc]

**Stable Odor Recognition by a neuro-adaptive Electronic Nose**

Authors:

*Eugenio Martinelli^1^, Gabriele Magna^1^, Davide Polese^1^, Alexander Vergara^2^, Detlev Schild^3,4 *^, Corrado Di Natale^1 *^*

1) Department of Electronic Engineering, University of Rome Tor Vergata, Via del Politecnico 1, Rome 00133, Italy

2) BioCircuits Institute, University of California San Diego, 9500 Gilman Dr., La Jolla, CA 92093-0402, USA

3) Inst. of Neurophysiology and Cellular Biophysics, University of Göttingen, Humboldtallee 23, 37077 Göttingen, Germany .

4) DFG Excellence Cluster 171 and Bernstein Forum of Neurotechnology, Univ. Göttingen

**SUPPLEMENTARY INFORMATION**


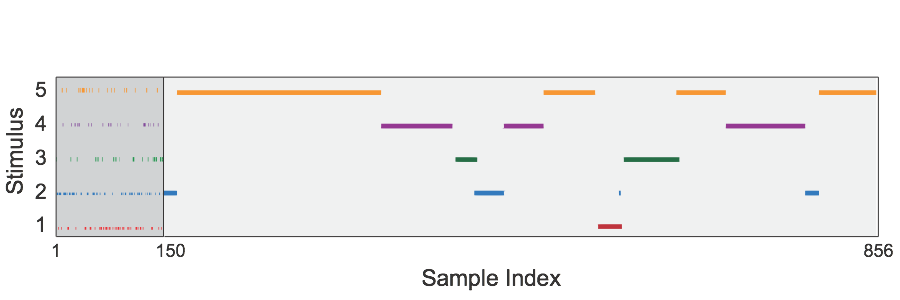


**Supplementary Figure 1.** Scheme of stimulus sequence. The five stimuli used are drawn in different colors and plotted over the course of the experiment. Random occurrences of stimuli, at high rate during the training phase of the network.


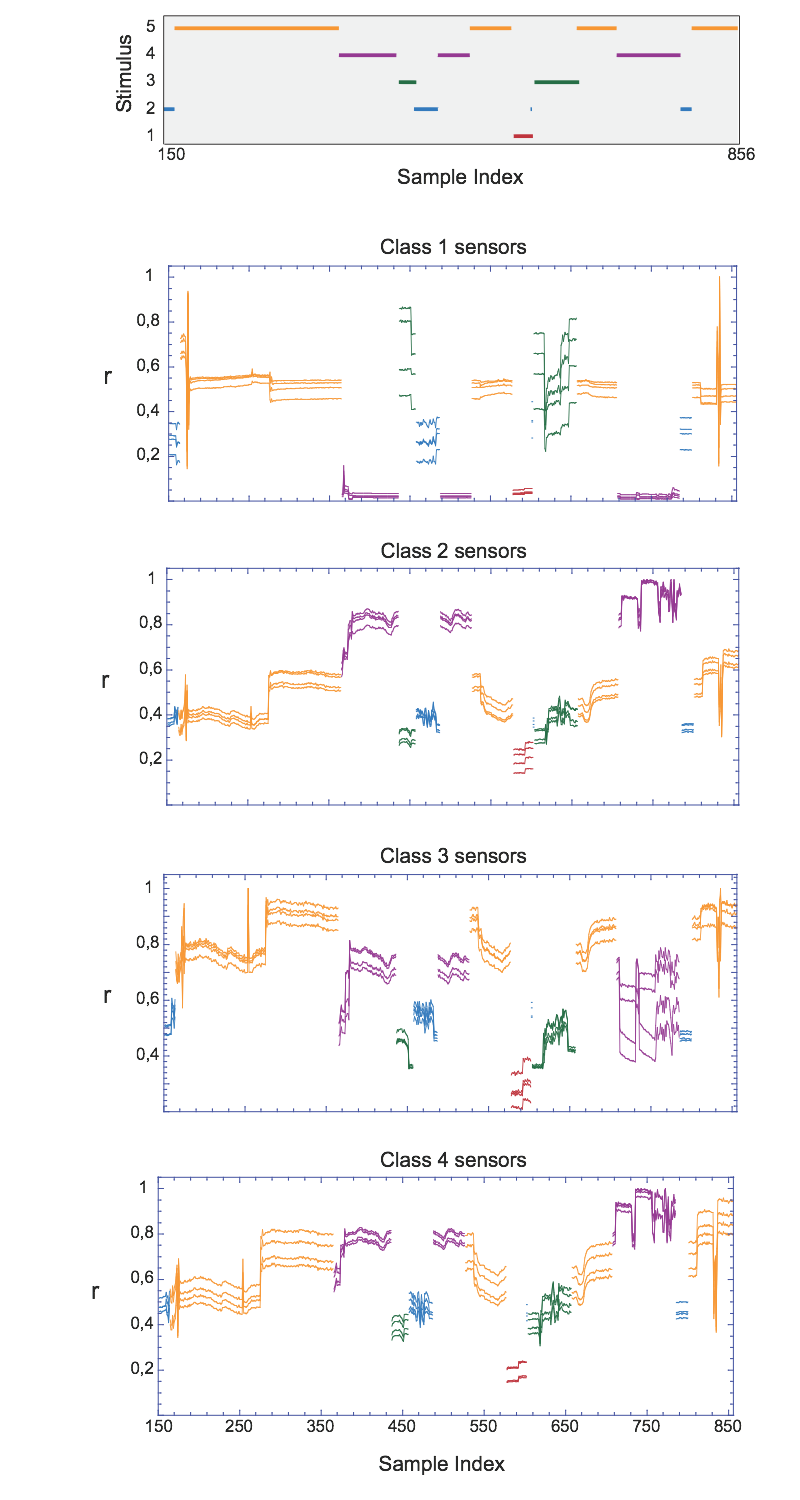


**Supplementary Figure 2.** Sensor responses of all sensor classes to the five stimuli used. Upper panel, scheme of stimulus application sequence; lower panels, responses of the sensor classes. Curves are normalized to the (min-max) - response range of the training phase.


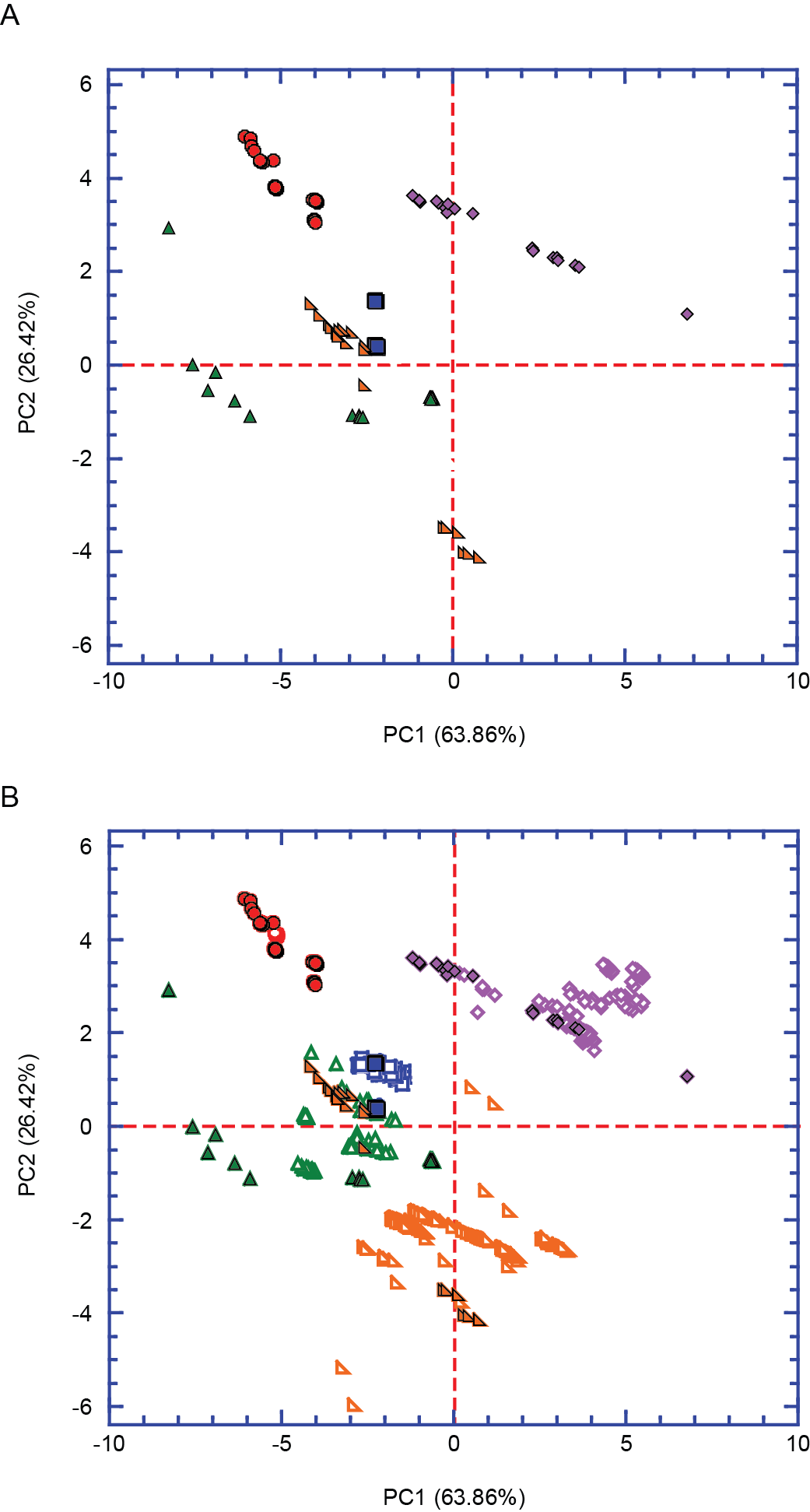


**Supplementary Figure 3.** Principal component analysis of the 16 sensor outputs. Plotted are the first two principal components for the training data (A) and training and testing data (B). Stimulus 1 through 5 are drawn in red, blue, green, violett, and orange, respectively. Closed and open symbols refer to the training phase (sample index 1 to 150) and the measurements after training, respectively.


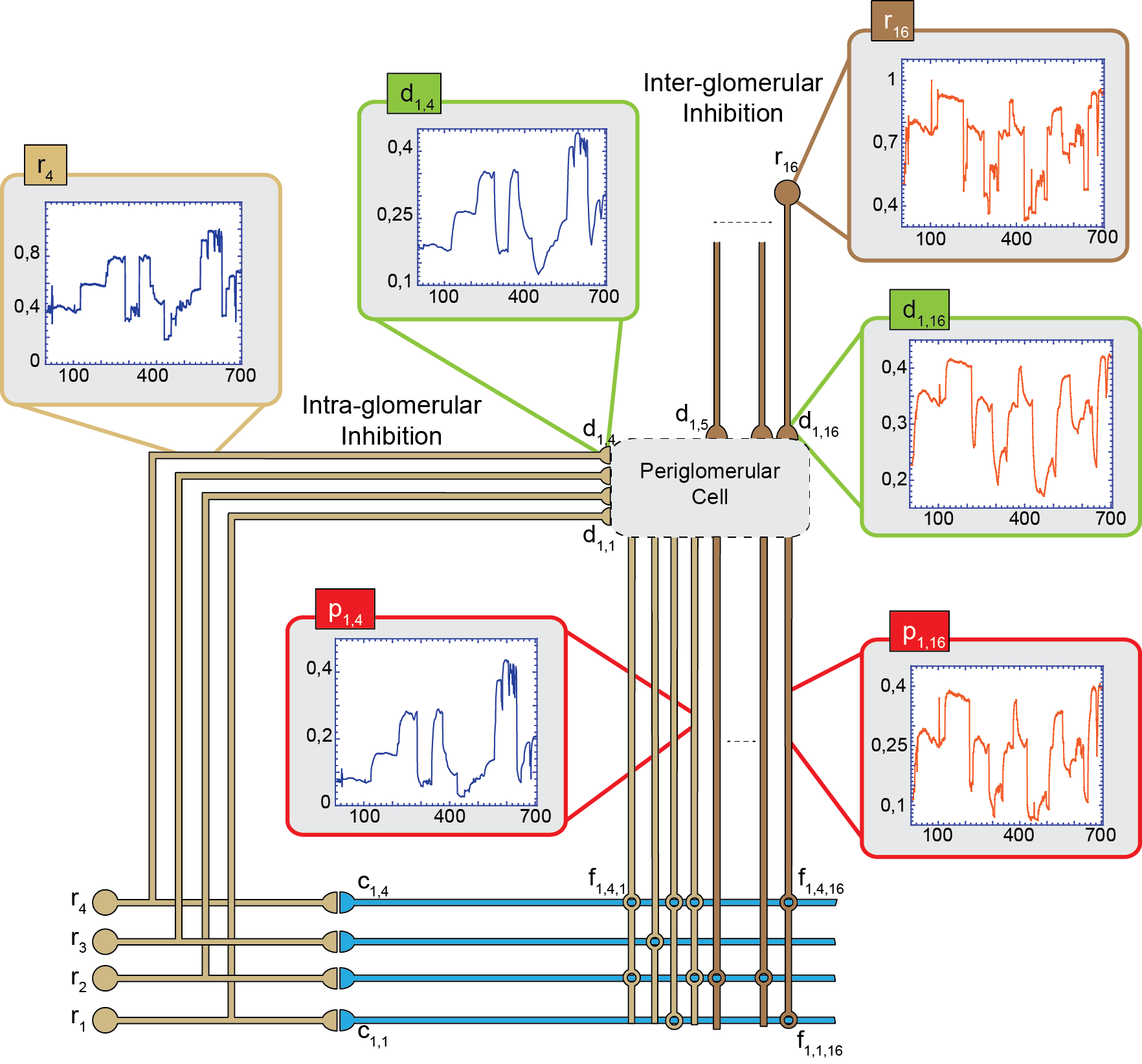
 **Supplementary Figure 4.** An example of intra- and inter-glomerular connections and signals of a PG cell for the given sequence of stimuli.
